# Association between body fat distribution and kidney stones: Evidence from a US population

**DOI:** 10.3389/fendo.2022.1032323

**Published:** 2022-10-07

**Authors:** Guoxiang Li, Hu Liang, Yunwu Hao, Qingfeng Huang, Xudong Shen, Yang Chen, Mingwei Chen, Junhua Xi, Zongyao Hao

**Affiliations:** ^1^ Department of Urology, The First Affiliated Hospital of Anhui Medical University, Hefei, China; ^2^ Institute of Urology, Anhui Medical University, Hefei, China; ^3^ Anhui Province Key Laboratory of Genitourinary Diseases, Anhui Medical University, Hefei, China; ^4^ Department of Endocrinology, The First Affiliated Hospital of Anhui Medical University, Hefei, China; ^5^ Department of Urology, The Second people’s Hospital of Hefei (Hefei Hospital Affiliated to Anhui Medical University), Hefei, China

**Keywords:** kidney stones, Android to Gynoid ratio, obesity, fat distribution, DXA

## Abstract

**Objectives:**

We aimed to evaluate the relationship between the proportion of Android to Gynoid ratio and the incidence of kidney stones among US adults.

**Methods:**

Participants aged 20-59 years from the 2011-2018 National Health and Nutrition Examination Survey (NHANES) database were selected to assess the association between Android to Gynoid ratio and kidney stone prevalence using logistic regression analysis, subgroup analysis and calculation of dose-response curves.

**Results:**

This study ultimately included 10858 participants, of whom 859 self-reported a history of kidney stones. And after adjusting for all confounders, an increased Android to Gynoid ratio was associated with an increased prevalence of kidney stones (OR=2.75, 95% CI:1.62-4.88). And subgroup analysis showed an increased prevalence of kidney stones in women (OR=3.55, 95% CI: 1.54-8.22), non-diabetic (OR=2.59, 95% CI: 1.45-4.60), 60 > age ≥ 40 years (OR=3.51, 95% CI: 1.83-6.71), Mexican-American (OR=4.35, 95% CI: 1.40- 13.53) and white (OR=3.86, 95% CI: 1.82-8.18) groups, there was a significant positive association between A/G ratio and kidney stones. In contrast, in the hypertensive subgroup, the A/G ratio was associated with kidney stones in all groups.

**Conclusions:**

Higher Android to Gynoid ratio is associated with a high prevalence of kidney stone disease.

## Introduction

Kidney stones disease (KSD) are among the most common and common diseases in urology and are caused by the abnormal accumulation of certain crystalline substances (such as calcium oxalate, calcium phosphate, uric acid, and drugs) in the kidney and are characterized by high prevalence and easy recurrence (1–3). A study based on data from the National Health and Nutrition Examination Survey (NHANES) reported that the prevalence of self-reported kidney stones in the United States was 11%, and the prevalence was 2% (4), which is an approximately 2.5-fold increase from the national prevalence (3.2%) in 1980 (5). Despite the fact that there are many treatment options for kidney stones, including extracorporeal shock wave lithotripsy (ESWL), rigid or flexible ureteroscopic stone extraction (URS/RIRS), and percutaneous nephrolithotripsy (PCNL), there is no single therapy that can cure them completely. The recurrence rate of kidney stones is 11% at two years, about 20% at five years, and up to 60% at five years in patients with recurrent attacks (6, 7). Without timely and effective treatment, kidney stones may cause extremely serious consequences such as permanent kidney damage and end-stage renal disease (8, 9). Furthermore, the costs associated with stone disease have risen significantly, with one study showing that kidney stone costs increased from approximately $2 billion in 2000 to over $3.79 billion in 2007 (10). Kidney stones have now become a very serious public health problem. Therefore it is of critical importance to investigate the risk factors for kidney stones and to take appropriate measures to prevent their occurrence.

Obesity has now become one of the serious health problems affecting the health of the global population (11, 12). Obesity can increase the risk of KSD (13, 14). Nevertheless, previous studies have primarily used body mass index (BMI) to assess obesity. Although BMI data are readily available and easy to calculate, they do not distinguish between adipose tissue, muscle tissue, and the distribution of adipose tissue throughout the body and are subject to inter- and intra-examiner variations. Sometimes even contradictory results are obtained (15). The reason may be that adults with similar BMIs have different fat distributions (16), and different fat distributions may have different health implications (17). For example, a lower risk of cardiometabolic dysfunction was observed in patients with gynoid fat distributions (characterized by preferential fat deposition in the buttocks and thighs, also referred to as pear patterns) compared to people with Android patterns (characterized by increased fat deposited in the trunk region, also referred to as apple patterns) (18).

For measuring body fat content and distribution, computed tomography (CT) and magnetic resonance imaging (MRI) are often considered the gold standard. However, the high radiation produced by CT, the lengthy acquisition and analysis times associated with MRI, and the higher costs of both techniques have limited their use in clinical and research settings. Dual-energy X-ray absorptiometry (DXA), on the other hand, is also sufficiently accurate and involves less radiation exposure, a shorter scan time, and a lower cost (19–21). Furthermore, DXA is well correlated with CT/MRI for measuring fat mass (FM) (19). Therefore, DXA measurements are increasingly being used in studies to assess the connection between obesity and a range of diseases (20, 22, 23).

An Android to Gynoid ratio (A/G ratio) is a DXA-based fat distribution index. Numerous studies have shown that the A/G ratio is strongly associated with insulin resistance and cardiovascular disease (24, 25), both of which are risk factors for KSD (26–28). Nevertheless, it remains unclear whether this potentially different fat distribution affects the prevalence of KSD. Therefore, in the present study, we aimed to assess the relationship between the A/G ratio and the prevalence of KSD in the United States (US) population.

## Materials and methods

### Study population

**Figure 1:**
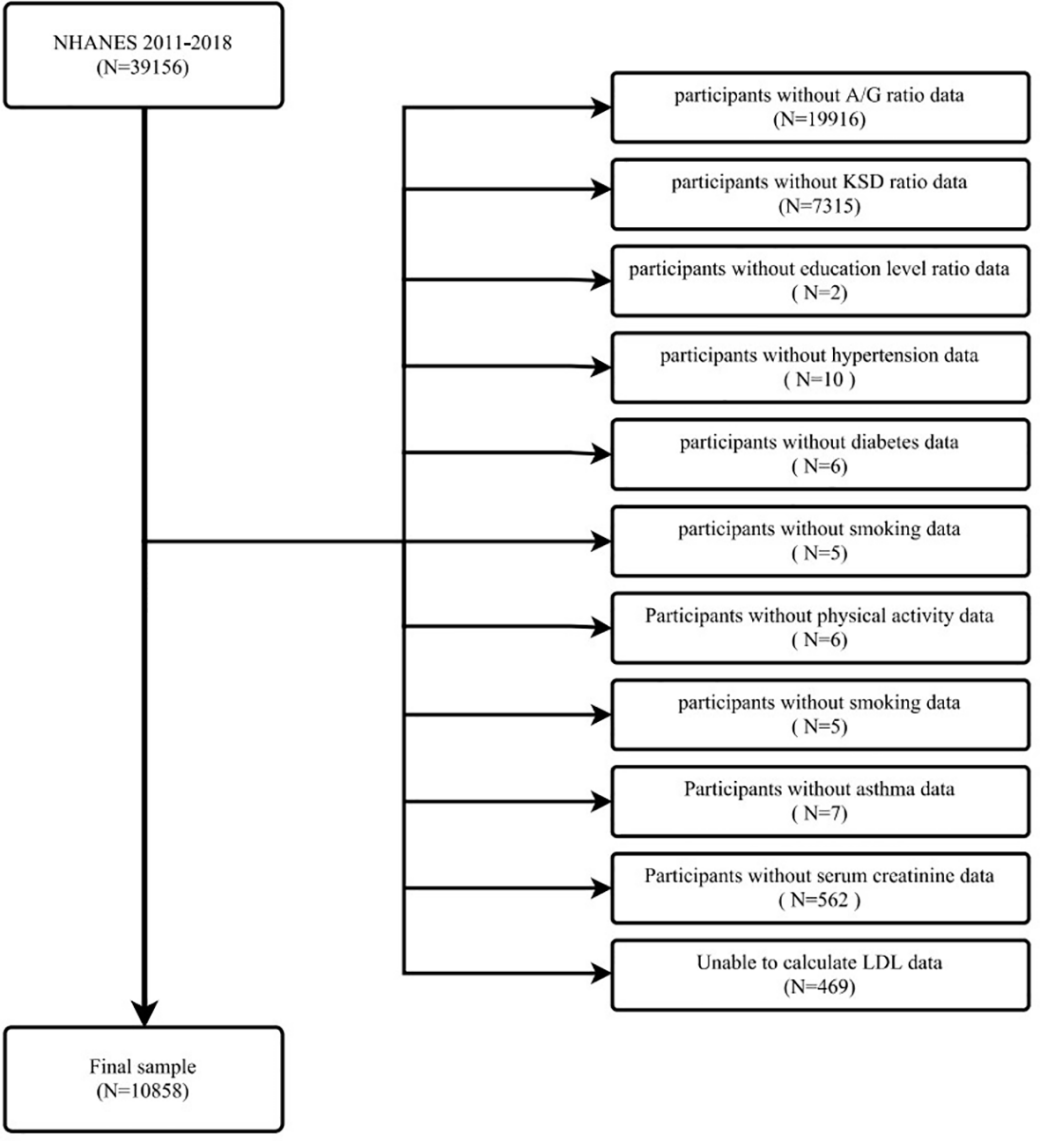
The participants selecting flow chart.

Data for the evaluation of this study were obtained from NHANES from 2011 to 2018. This is a survey conducted by the Centers for Disease Control and Prevention (CDC) every two years for public health surveillance in the U.S. The NHANES study protocol was reviewed and approved by the Institutional Review Board of the National Center for Health Statistics (NCHS), and all participants provided written informed consent. All methods were conducted in accordance with relevant guidelines and regulations. Our study examined data from four consecutive two-year survey cycles. All participants were evaluated with the KIQ026 survey (Do you have kidney stones) and a total of 39,156 people participated in the questionnaire. The exclusion criteria are shown in the figure (**Figure 1**). In total, 10858 cases were included in this study, of which 859 had a self-reported history of kidney stones.

### Data collection and definition

A/G ratios (Variable Name: DXXAGRAT) were designed as exposure variables. Relevant data were obtained by performing DXA measurements on subjects. DXA examinations were obtained by trained and certified radiologic technologists using a Hologic Discovery Model A densitometer (Hologic, Inc., Bedford, Massachusetts). The examination excluded subjects who were pregnant (urine pregnancy test and/or self-reported positive at the time of the DXA examination), self-reported a history of X-ray contrast (barium) use within the past seven days, or measured more than 450 pounds or more than 6’5” (DXA table limit). Whole-body scans were obtained on a Hologic Discovery A densitometer (Hologic, Inc., Bedford, MA) using software version Apex 3.2. The Android and Gynoid regions were determined by the HOGIC APEX software used in the scan analysis for automatic delineation (29). Finally, the Android/Gynoid ratio was calculated based on the measured Android (Variable Name: DXXANFM) and Gynoid (Variable Name: DXXGYFM) data. For more information, please refer to: https://wwwn.cdc.gov/Nchs/Nhanes/2017-2018/DXXAG_J.htm. The occurrence of kidney stones (Variable Name: KIQ026) was designed as an outcome variable. The questionnaire KIQ026 (ever had kidney stones)? was used to assess kidney stones; if the participant answered “yes,” he was considered to have kidney stones.

In order to summarize potential confounders that could confound the relationship between A/G ratio and KSD, adjusted multivariate models were used. The following covariates were selected: age, gender, race, education level, poverty to income ratio (PIR), marital status, alcohol consumption, smoking status, physical activity, METS-IR, BMI, diabetes, hypertension, asthma, laboratory tests (cholesterol level, serum creatinine, blood calcium, blood phosphorus, blood uric acid, cholesterol, triglyceride, HDL, LDL, glycosylated hemoglobin) and some dietary intake factors (total energy intake, total fat intake, total sugar intake and total water intake). A 24-hour dietary recall was completed by all participants, and the average intake of the two recalls will be used in the analysis. The dietary intake factors had a high number of missing values, which we converted to categorical variables. We assessed these variables in tertile, with the lowest tertile serving as the reference group and missing values set as dummy variables. These covariates were determined using self-report questionnaires, interviews, physical examinations, and laboratory measurements. Information on age, gender, race/ethnicity, PIR, smoking, alcohol consumption, history of hypertension, and history of diabetes were determined by questionnaire. Information on the dietary intake of the participants was obtained through interviews. Participants’ BMI and waist circumference were obtained by physical examination, and laboratory measurements were used for the remaining covariates. Cholesterol, triglyceride, and uric acid concentrations in serum or plasma were measured using the timed-endpoint method. The timed rate biuret method was used to measure phosphorus in blood, and the indirect (or diluted) I.S.E. (ion selective electrode) method was used to measure calcium concentrations in serum, plasma, or urine (uses indirect (or diluted) I.S.E. (ion selective electrode) methodology to measure calcium concentration, the modular chemistry side uses the Jaffe rate method (kinetic alkaline picrate) to determine serum, plasma, or urine Creatinine concentrations in creatinine, Tosoh Automated Glycohemoglobin Analyzer HLC-723G8 was used to measure glycosylated hemoglobin in patients, HDL data were obtained using Roche/Hitachi Cobas 6000 Analyzer, patients with excess LDL deficiency, we used Friedewald formula to recalculate the patient’s LDL. Details of the study variables used are all publicly available at www.cdc.gov/nchs/nhanes/.

### Statistical methods

To illustrate the complex, multi-stage sampling design used to select a representative non-institutionalized U.S. population, the sampling weights, strata, and subgroups provided in the NHANES study were applied to all statistical analyses. Continuous variables are expressed as weighted means and 95% CIs, and categorical variables are expressed as weighted proportions and 95% CI. We first did a VIF covariate screening of all covariates and removed the covariate if the VIF value was greater than five, considered to have sharedness. Based on the guidelines (30), three different logistic regression models were used to examine the relationship between the A/G ratio and KSD. In model 1, no covariates were adjusted. Model 2 was adjusted for age, gender and race, marital status, and education level, while Model 3 was adjusted for age, gender and race, marital status, education level, alcohol consumption, smoking status, physical activity, METS-IR, diabetes, hypertension, asthma, cholesterol level, serum creatinine, blood calcium, blood phosphorus, blood uric acid, cholesterol, triglyceride, HDL, LDL, glycosylated hemoglobin, total energy intake, total fat intake, total sugar intake and total water intake. A smoothed curve fit (penalized spline method) and generalized additive model (GAM) regression were then performed to further assess the relationship between A/G ratio and KSD. Multiple regression analysis was then conducted stratified by age, gender, race, diabetes, and hypertension mellitus. Moreover, interaction terms were added using a log-likelihood ratio test to test for heterogeneity in the association between subgroups. P < 0.05 was considered statistically significant. All analyses were performed using Empower software (www.empowerstats.com; X&Y Solutions, Inc., Boston, MA, USA) and R version 3.4.3 (http://www.R-project.org, The R Foundation).

## Results

### Characteristics of the study population

The analysis involved 11327 participants, including 895 patients with kidney stones (**Table 1**). In the stone group, A/G ratio was significantly higher than in the non-stone group (1.05 > 0.99, p<0.0001).

**Table 1:** The characteristics of the participants selected.

Characteristic	Nonstone formers	Stone formers	P-value
	N=9999	N=859	
Age	39.23 (38.74,39.72)	43.51 (42.47,44.56)	<0.0001
Serum Calcium (MG/DL)	9.37 (9.36,9.38)	9.34 (9.30,9.39)	0.2245
Serum Creatinine (MG/DL)	0.86 (0.85,0.86)	0.87 (0.84,0.90)	0.3303
Cholesterol (MG/DL)	190.63 (189.25,192.00)	194.20 (190.48,197.92)	0.0536
Serum phosphorus (MG/DL)	3.72 (3.70,3.74)	3.65 (3.59,3.71)	0.0228
Uric acid (MG/DL)	5.32 (5.28,5.36)	5.38 (5.27,5.50)	0.2692
HDL(MG/DL)	53.79 (53.21,54.38)	50.34 (48.90,51.78)	<0.0001
HBA1C(MG/DL)	5.49 (5.46,5.51)	5.71 (5.61,5.81)	0.0001
LDL(MG/DL)	110.32 (109.22,111.42)	113.97 (110.76,117.17)	0.0318
Triglyceride (MG/DL)	132.59 (129.90,135.27)	149.45 (142.59,156.32)	<0.0001
METS-IR	42.43 (41.94,42.92)	46.93 (45.67,48.19)	<0.0001
BMI	28.72 (28.46,28.98)	30.81 (30.15,31.48)	<0.0001
Waistline	97.39 (96.77,98.02)	103.31 (101.92,104.70)	<0.0001
Android to Gynoid ratio	0.99 (0.99,1.00)	1.05 (1.03,1.07)	<0.0001
Android fat mass	2425.66 (2375.35,2475.97)	2943.12 (2817.65,3068.58)	<0.0001
Gynoid fat mass	4711.65 (4648.13,4775.16)	5067.54 (4883.02,5252.07)	0.0003
Visceral adipose tissue volume	526.75 (515.08,538.42)	699.65 (668.59,730.71)	<0.0001
Total fat	27187.60 (26815.40,27559.80)	29957.26 (29056.16,30858.35)	<0.0001
Gender			0.4968
Male	50.57 (49.35,51.78)	48.75 (43.76,53.77)	
Female	49.43 (48.22,50.65)	51.25 (46.23,56.24)	
Race			<0.0001
Mexican American	17.01 (14.44,19.92)	14.30 (11.02,18.35)	
White	61.25 (57.42,64.94)	72.05 (66.55,76.96)	
Black	12.14 (10.16,14.45)	6.71 (5.16,8.69)	
Other Race	9.61 (8.50,10.84)	6.94 (5.14,9.30)	
Education Level (%)			0.7453
Less than high school	18.63 (16.56,20.90)	17.78 (14.97,20.98)	
High school	30.43 (28.40,32.55)	32.22 (27.05,37.86)	
More than high school	50.93 (47.97,53.89)	50.01 (44.70,55.31)	
Marital Status (%)			0.0015
Cohabitation	61.84 (59.90,63.74)	67.75 (63.66,71.59)	
Solitude	38.16 (36.26,40.10)	32.25 (28.41,36.34)	
PIR			0.4507
<1.39	21.57 (19.61,23.68)	20.71 (18.06,23.64)	
1.39-3.49	31.76 (29.81,33.78)	35.24 (30.59,40.20)	
≥3.49	39.84 (36.94,42.81)	38.34 (33.36,43.58)	
Unclear	6.83 (5.91,7.87)	5.70 (3.85,8.35)	
Alcohol (%)			0.6265
Yes	60.94 (58.62,63.20)	61.01 (56.01,65.79)	
No	15.95 (14.40,17.64)	17.37 (13.71,21.76)	
Unclear	23.11 (21.34,24.99)	21.62 (17.47,26.43)	
Smoked			0.015
Yes	40.65 (38.89,42.43)	46.57 (41.80,51.39)	
No	59.35 (57.57,61.11)	53.43 (48.61,58.20)	
Hypertension (%)			<0.0001
Yes	21.22 (19.99,22.51)	36.89 (32.56,41.45)	
No	78.78 (77.49,80.01)	63.11 (58.55,67.44)	
Diabetes (%)			<0.0001
Yes	5.00 (4.48,5.57)	11.92 (9.31,15.14)	
No	95.00 (94.43,95.52)	88.08 (84.86,90.69)	
Physical Activity (%)			0.0795
Never	20.87 (19.78,22.02)	25.06 (21.37,29.16)	
Moderate	28.37 (27.03,29.74)	26.97 (23.42,30.84)	
Vigorous	50.76 (49.27,52.25)	47.97 (43.44,52.53)	
Asthma			0.1986
No	84.39 (83.25,85.47)	82.62 (79.81,85.11)	
Yes	15.61 (14.53,16.75)	17.38 (14.89,20.19)	
Total Kcal (%)			0.2265
Tertile 1	26.56 (25.19,27.98)	30.26 (26.08,34.81)	
Tertile 2	28.77 (27.48,30.10)	29.06 (24.77,33.76)	
Tertile 3	29.40 (28.14,30.70)	25.50 (21.65,29.79)	
Unclear	15.26 (14.20,16.39)	15.17 (12.34,18.52)	
Total Sugars (%)			0.6518
Tertile 1	24.26 (23.08,25.49)	26.51 (22.58,30.85)	
Tertile 2	24.34 (23.15,25.56)	22.65 (19.51,26.13)	
Tertile 3	23.91 (22.62,25.26)	23.46 (20.20,27.06)	
Unclear	27.49 (26.19,28.82)	27.38 (23.66,31.44)	
Total Water (%)			0.6317
Tertile 1	25.94 (24.68,27.24)	26.71 (22.92,30.88)	
Tertile 2	28.99 (27.80,30.21)	30.75 (27.04,34.73)	
Tertile 3	29.81 (28.49,31.17)	27.36 (23.38,31.74)	
Unclear	15.26 (14.20,16.39)	15.17 (12.34,18.52)	
Total Fat (%)			0.6317
Tertile 1	25.94 (24.68,27.24)	26.71 (22.92,30.88)	
Tertile 2	28.99 (27.80,30.21)	30.75 (27.04,34.73)	
Tertile 3	29.81 (28.49,31.17)	27.36 (23.38,31.74)	
Unclear	15.26 (14.20,16.39)	15.17 (12.34,18.52)	
			

### Elevated A/G ratio was associated with increased prevalence of kidney stones

VIF values for all covariates for covariate screening were less than 5, so all covariates were included in the final model. A multifactorial logistic regression analysis was performed showing a 1.75-fold increase in the prevalence of kidney stones for each unit increase in the A/G ratio (OR=2.75, 95% CI:1.62-4.88) (**Table 2**). We then converted the A/G ratio from a continuous variable to a categorical variable (triplet). The results showed a 41% higher likelihood of kidney stones in the highest tertile (tertile 3) compared to the lowest A/G ratio in the lowest tertile (tertile 1), as shown in **Table 2**.

**Table 2:** Analysis between A/G ratio with kidney stone formation.

Characteristic	Model 1 OR (95%CI)	Model 2 OR (95%CI)	Model 3 OR (95%CI)
A/G ratio	4.17 (2.89, 6.02)	5.04 (3.25, 7.82)	2.75 (1.62, 4.68)
Categories			
Tertile 1	1	1	1
Tertile 2	1.17 (0.95, 1.45)	1.22 (0.98, 1.52)	0.99 (0.78, 1.26)
Tertile 3	1.85 (1.51, 2.28)	2.01 (1.59, 2.52)	1.41 (1.07, 1.85)
P for trend	<0.001	<0.001	0.002

Model 1 = no covariates were adjusted.

Model 2 = Model 1+age, gender, race, education, marital status were adjusted.

Model 3 = Model 2+age, gender and race, marital status, education level, alcohol consumption, smoking status, physical activity, METS-IR, diabetes, hypertension, asthma, cholesterol level, serum creatinine, blood calcium, blood phosphorus, blood uric acid, cholesterol, triglyceride, HDL, LDL, glycosylated hemoglobin, total energy intake, total fat intake, total sugar intake and total water intake were adjusted.

### Subgroup analysis

To assess the robustness of the association between the A/G ratio and the prevalence of kidney stones, a subgroup analysis was performed (**Table 3**). The results showed that in the hypertensive subgroup, all increases in the A/G ratio were positively associated with the prevalence of kidney stones. In contrast, in the diabetic subgroup, only the non-diabetic group had a significant positive association of A/G ratio with kidney stones (OR=2.59, 95% CI: 1.45-4.60). In the age subgroup, a significant positive association between A/G ratio and kidney stones was found only in the group of 40 ≤ age < 60 (OR=3.51, 95% CI: 1.83-6.71). Among the ethnic subgroups, a significant positive association between A/G ratio and kidney stones was found in Mexican Americans (OR=4.35, 95% CI: 1.40-13.53) and Whites (OR=3.86, 95% CI: 1.82-8.18). In the gender subgroup, a significant positive association between A/G ratio and kidney stones was found only in the female group (OR=3.55, 95% CI: 1.54-8.22). In addition, we tested for interactions with age, sex, race, hypertension, and diabetes mellitus. However, no correlations were detected with interactions meeting statistical significance (p > 0.05 for all interactions)

**Table 3:** Subgroup analysis between A/G ratio with kidney stone formation.

Characteristic	Model 1	Model 2	Model 3	P for trend*	P for Interaction*
Gender					0.4189
Male	7.38 (4.22, 12.91)	3.77 (2.07, 6.85)	1.97 (0.98, 3.99)	0.192	
Female	6.66 (3.45, 12.88)	6.36 (3.27, 12.37)	3.55 (1.54, 8.22)	0.006	
Race					0.1285
Mexican American	3.33 (1.54, 7.21)	3.72 (1.42, 9.76)	4.35 (1.40, 13.53)	0.0126	
White	5.07 (3.05, 8.42)	7.66 (4.18, 14.02)	3.86 (1.82, 8.18)	0.001	
Black	2.40 (0.86, 6.69)	1.73 (0.54, 5.60)	1.32 (0.29, 5.92)	0.753	
Others	6.15 (2.16, 17.50)	5.31 (1.43, 19.77)	1.40 (0.30, 6.60)	0.697	
Diabetes					0.4508
Yes	2.85 (0.93, 8.68)	4.58 (1.19, 17.62)	4.30 (0.95, 19.37)	0.181	
No	3.64 (2.45, 5.41)	4.41 (2.75, 7.07)	2.59 (1.45, 4.60)	0.008	
Hypertension					0.8256
Yes	2.53 (1.34, 4.78)	3.97 (1.85, 8.51)	3.59 (1.49, 8.64)	0.073	
No	3.56 (2.23, 5.68)	4.06 (2.34, 7.07)	2.28 (1.15, 4.51)	0.029	
Age					0.0881
<40	1.97 (1.03, 3.77)	3.52 (1.70, 7.30)	1.72 (0.64, 4.63)	0.382	
40-59	4.09 (2.60, 6.45)	6.24 (3.58, 10.88)	3.51 (1.83, 6.71)	<0.001	
					

Model 1 = no covariates were adjusted.

Model 2 = Model 1+age, gender, race, education, marital status were adjusted.

Model 3 = Model 2+age, gender and race, marital status, education level, alcohol consumption, smoking status, physical activity, METS-IR, diabetes, hypertension, asthma, cholesterol level, serum creatinine, blood calcium, blood phosphorus, blood uric acid, cholesterol, triglyceride, HDL, LDL, glycosylated hemoglobin, total energy intake, total fat intake, total sugar intake and total water intake were adjusted.

*Means only in model 3.

### Analysis of dose-response and threshold effects of A/G ratio on the prevalence of KSD

**Figure 2:**
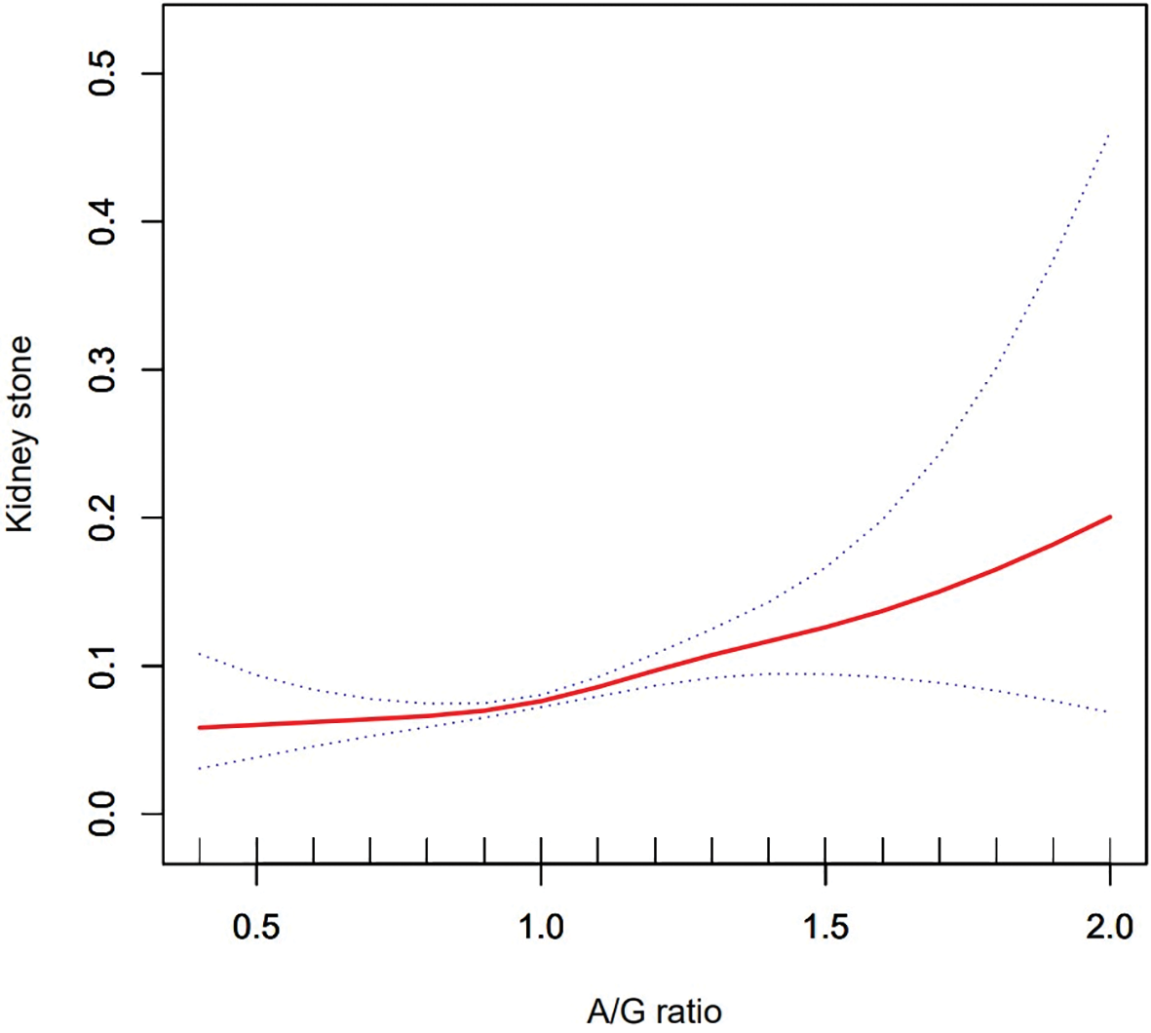
Density dose–response relationship between Android to Gynoid ratio with kidney stone formation. The area between two dotted lined is expressed as a 95% CI.

Using a generalized additive model and smoothed curve fitting, a relationship between A/G ratio and kidney stones was further investigated. Our results show that the A/G ratio is linearly and positively correlated with the KSD (**Figure 2**).

## Discussion

KSD has a complex etiology, a high recurrence rate, and a wide range of individual variations. Therefore, examining KSD’s risk factors is essential for both prevention and treatment. According to our knowledge, this is the first study to investigate the association between A/G ratios and the prevalence of nephrolithiasis. According to our analysis of four consecutive NHANES two-year cycles (2011–2018) of a nationally representative population, the A/G ratio is predictive of kidney stone occurrence, and the higher the A/G ratio, the higher the risk.

This correlation may be due to a number of reasons. First, an increase in the A/G ratio generally represents an abnormal increase in fat content in the Android region. Android region contains the liver, pancreas, and kidneys. There have been numerous studies demonstrating that fat accumulation in these structures can have harmful effects both directly and indirectly on the body (31–33). An accumulation of fat in the liver and pancreas is associated with multiple indicators of inflammation (34), and inflammation has been strongly linked to kidney stone formation (35). The accumulation of fat in and around the kidneys may have a significant impact on kidney function and blood pressure (36). A study in obese animals found that increased renal sinus fat may increase blood pressure and kidney interstitial pressure by compressing the blood vessels that leave the kidney (37). Local ischemia may also result from this phenomenon, which can result in renal tubular injury (38). Lipid accumulation within the kidney parenchyma, in turn, can result in lipotoxicity, inflammation, oxidative stress, and kidney fibrosis (39, 40). These factors have been shown to be risk factors for KSD in numerous studies (35, 41, 42). In addition, the gynoid region contains the buttocks and part of the thighs. Adipose tissue in this region is generally considered to have a health-promoting effect (43), and an increase in the ratio may indicate that the damaging factors begin to outweigh the protective factors, leading to the development of KSD. Second, both KSD and A/G ratio are closely related to intestinal flora. Studies have shown that increased abundance of Faecalibacterium prausnitzii in the intestine of obese adolescents who underwent fecal transplantation was associated with a lower A/G ratio (44), whereas calcium oxalate stones were negatively associated with the abundance of Faecalibacterium spp (45). Therefore, the correlation between high A/G ratio and increased prevalence of KSD may also be mediated by changes in the abundance or species of intestinal flora. This requires further study. Third. Studies have shown that the lower the serum carotenoid content, the higher the A/G ratio in Chinese (46). In addition, the higher prevalence of kidney stones is also positively correlated with low serum carotenoid content (47). Finally, it has been shown that higher Android/Gynoid ratios are associated with steady-state model assessment of insulin resistance (HOMA-IR) values, metabolic syndrome (METS), nonalcoholic fatty liver disease (NAFLD), and triglyceride glycemic index levels (22, 48, 49), which are also risk factors for KSD (26, 50–52), were also positively correlated, which may account for the association of KSD with the A/G ratio.

We also stratified the variables by age, sex, race, hypertension, and diabetes status in this study. There was a positive correlation between the prevalence of the A/G ratio and the KSD in all subgroups when unadjusted. After adjusting for all variables, this correlation was statistically significant in all groups only if they were grouped according to hypertension. However, the correlation between A/G ratio and KSD was more significant in the hypertensive group. This may be because hypertension itself is a risk factor for KSD (53), so it enhances this correlation. Interestingly, diabetes itself is also a risk factor for KSD. However, in the diabetes subgroup, the correlation between A/G ratio and KSD was lost in the diabetic group. This may be because several glucose-lowering drugs can prevent kidney stone formation. For example, metformin prevents kidney stone formation by attenuating oxalic acid-induced lipid peroxidation products-induced tubular damage and by inhibiting the expression of osteopontin (OPN) and monocyte chemoattractant protein 1 (MCP-1) (54, 55). Rosiglitazone may also inhibit renal crystal deposition by ameliorating tubular damage due to oxidative stress and inflammatory responses through multiple pathways (56, 57). Another possible explanation is that a high-sugar, high-fat diet is more likely to lead to metabolic syndrome (58), whereas diabetic patients are generally more conscious of dietary management and are relatively more protective, which may be more beneficial in preventing KSD formation. Furthermore, when stratified by gender, the relationship between A/G ratio and KSD was statistically significant only in the female population. This is similar to previous studies on A/G ratios. This may be due to the fact that the fat distribution in the female group is dominated by Gynoid pattern fat distributions. Thus, the female group may accumulate relatively more fat in the Android region before showing an increase in the A/G ratio (59), this may have a greater impact on the body. Sex hormones may also play an important role, with sex hormones shifting to androgen production as Android body fat increases (60).

However, after adjusting for all confounding variables. This correlation was only significant among Caucasians and Mexican-Americans in stratification by race. It may be due to the fact that black groups appear to be less affected by obesity than other races (61). Further, in stratification by age, a significant positive association was found between the A/G ratio and the prevalence of KSD only in groups older than 40 years of age. This is probably because aging adipose tissue promotes insulin resistance and lipid penetration (62, 63). In addition, aging reduces the ability of adipose tissue to store free fatty acids, causing a lipotoxic environment and systemic lipotoxicity (64). This, in turn, leads to kidney damage, which in turn contributes to kidney stone formation (35, 42).

The study has several advantages. First of all, this is the first comprehensive analysis of the correlation between the A/G ratio and KSD. Second, NHANES follows a well-designed study protocol with extensive quality assurance and quality control. In addition, the large representative sample size makes our results more reliable and generalizable to the entire US multi-ethnic adult population. Furthermore, the wide range of covariates used for adjustment enhances the accuracy of statistical inferences.

Of course, our study has some limitations. First, our study was based on the NHANES database, which is a cross-sectional study, and we were unable to obtain a causal relationship between the A/G ratio and kidney stones. Second, out of the DXA test results, many of our data were based on self-reporting, which may have some recall and reporting bias, such that a small number of asymptomatic urinary stones may be excluded. Third, the database did not provide more detailed information, such as medication history and stone composition. It is therefore necessary to conduct further research in order to confirm our results and explore in more detail the correlation between the A/G ratio and KSD.

## Conclusion

Based on a cross-sectional study of a US population, we found that a high A/G ratio was associated with an increased prevalence of kidney stones. This may have significant implications for the prevention and treatment of kidney stones. Therefore, this needs to be validated by further studies and the potential mechanisms explored.

## Data availability statement

Publicly available datasets were analyzed in this study. This data can be found here: https://www.cdc.gov/nchs/nhanes/index.htm.

## Author contributions

GL, HL, and YH: Conceptualization, Methodology, Software. QH, XS, and YC: Visuali-zation, Investigation. MC, JX, and ZH: Writing - review & editing. All authors contributed to the article and approved the submitted version.

## Funding

This work was supported by the National Natural Science Foundation of China (82070724; 82000672).

## Acknowledgments

The authors are grateful for the invaluable support and useful discussions with other members of the urological department. We are also grateful to all participants and research teams in the National Health and Nutrition Examination Survey.

## Conflict of interest

The authors declare that the research was conducted in the absence of any commercial or financial relationships that could be construed as a potential conflict of interest.

## Publisher’s note

All claims expressed in this article are solely those of the authors and do not necessarily represent those of their affiliated organizations, or those of the publisher, the editors and the reviewers. Any product that may be evaluated in this article, or claim that may be made by its manufacturer, is not guaranteed or endorsed by the publisher.

## Abbreviations

NHANES, National Health and Nutrition Examination Survey; OR, Odds ratio; CI, Confidence interval; A/G ratios, Android to Gynoid ratio; DXA, Dual-energy X-ray absorptiometry; US, United States; PIR, Ratio of family income to poverty; KSD, Kidney stones disease; BMI, Body mass index.
